# Gross Ascites Secondary to Endometriosis: A Rare Presentation in Pre-Menopausal Women

**DOI:** 10.7759/cureus.17048

**Published:** 2021-08-10

**Authors:** Vishal Bahall, Lance De Barry, Suman S Harry, Maria Bobb

**Affiliations:** 1 Obstetrics and Gynaecology, The University of the West Indies, St Augustine, TTO; 2 Obstetrics and Gynaecology, San Fernando General Hospital, San Fernando, TTO; 3 Obstetrics and Gynaecology, Sangre Grande Hospital, Sangre Grande, TTO

**Keywords:** endometriosis related ascites, endometriosis, gross ascites, fertility, elevated ca-125

## Abstract

Ascites caused by endometriosis is an unusual phenomenon with approximately 60 cases described since it was first reported in 1954. Moreover, such a case has rarely been reported in the Caribbean literature. Ascites is frequently treated with surgical options that do not preserve fertility. This is due to the association of ascites with gynaecological malignancies in women with elevated serum cancer antigen (CA-125). We describe three cases of severe endometriosis associated with massive ascites, successfully treated with hormonal therapy while preserving fertility.

## Introduction

Endometriosis is a common condition affecting up to 10% of women of reproductive age [1]. It is a debilitating condition characterized by high recurrence rates and diagnosed in up to 30% of patients evaluated for infertility [2]. Defined as the presence of endometrial-like tissue (glands and stroma) outside the endometrial cavity, endometriotic deposits respond to hormonal stimuli and undergo cyclical bleeding [3]. Patients report symptoms of dysmenorrhoea, menorrhagia, chronic pelvic pain, and dyspareunia [4]. While up to 20-25% of patients may remain completely asymptomatic, one rare presentation of severe endometriosis is massive haemorrhagic ascites [3, 5]. 

Ascites secondary to endometriosis is found to be prevalent in nulliparous women of African descent [5, 6]. Ascites secondary to endometriosis constitutes a diagnostic dilemma due to its variations in presentation and location. This clinical entity may be easily mistaken for a malignancy such that a systematic review done in 2010 by Gungor et al. reported that ovarian malignancy was suspected in 52.4% of cases during diagnostic work-up [5]. Like ovarian cancer, ascites and elevated levels of serum CA-125 are also noted in severe endometriosis [7]. In this series, we describe three cases of severe endometriosis associated with progressive abdominal distension and massive ascites successfully managed with medical treatment after initial diagnosis, avoiding surgical menopause.

## Case presentation

Case 1

A 22-year-old nulliparous female of African descent presented with a four-day history of vomiting, abdominal pain and abdominal distension. She has a history of well-controlled asthma and was otherwise well. One year ago, she underwent an exploratory laparotomy and omental biopsy due to a similar presentation. During that procedure, three litres of ascitic fluid was drained and histological analysis of the biopsied specimen confirmed endometriosis with no evidence of malignancy.

**Figure 1:**
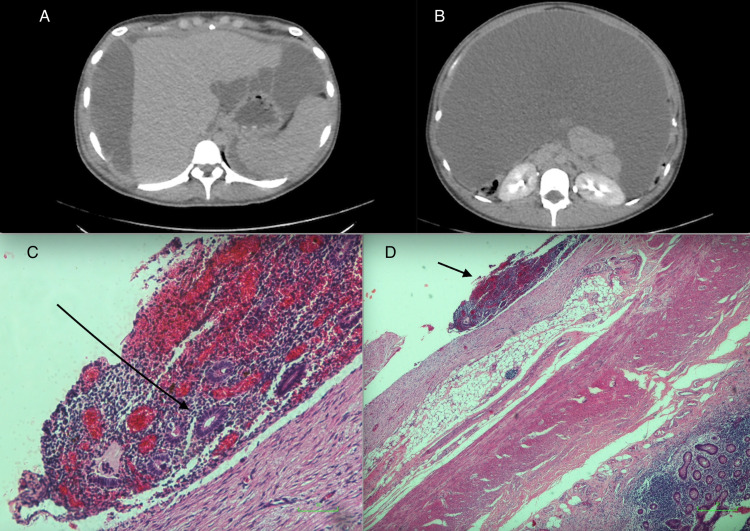
(A) and (B) Axial images demonstrating gross ascites within abdomen extending to liver. (C) Arrow shows endometriotic deposit on serosa. (D) Arrow shows endometrial glands with stroma typical of endometriosis

On this presentation, a computed tomography (CT) scan of the abdomen and pelvis showed tense ascites with scalloping of the liver consistent with pseudomyxoma peritonei (Figure 1). The intra-abdominal viscera appeared unremarkable. A 6.4cm x 3.1cm complex ovarian cyst with thin septations suggestive of mucinous ovarian neoplasm was noted and the uterus was grossly normal. No enlarged lymph nodes or suspicious bone lesions were identified. 

Abdominocentesis was performed and nine litres of ascitic fluid was drained. Cytology showed reactive mesothelial cells, a few mononuclear cells and atypical cells of unknown significance. She received a three monthly dose of gonadotropin-releasing hormone therapy (GnRH analogue). 

The patient subsequently underwent an exploratory laparotomy, where a further two litres of ascitic fluid was drained. The intra-operative findings were consistent with peritoneal endometriosis. Biopsies of the peritoneum and appendix were taken. Histological analysis of the peritoneum showed portions of necrotic fibro-collagenous tissue densely infiltrated by acute and chronic inflammatory cells as well as a population of hemosiderin-laden macrophages in keeping with recent haemorrhage (Figure 1). The appendix measured 5.5cm x 1.5cm and histology showed several deposits of endometriosis on the serosal surface composed of endometrial type glands (Figure 1) replete with appropriate stroma associated with chronic inflammation. Overall, features were consistent with peritoneal endometriosis. There was no evidence of malignancy on histopathology.

Post operatively, she was treated with a monthly dose of GnRH and placed on combined oral contraceptive pills. At routine follow up, there was no report of abdominal pain, further abdominal distension or ultrasonographic evidence of ascites. This patient is currently asymptomatic with no signs of recurrence.

Case 2

A 34-year-old East Indian female presented with a two-week history of abdominal distension. Two months ago, she underwent a laparoscopic cystectomy and left salpingectomy secondary to clinically severe endometriosis. She was otherwise healthy with no children.

**Figure 2:**
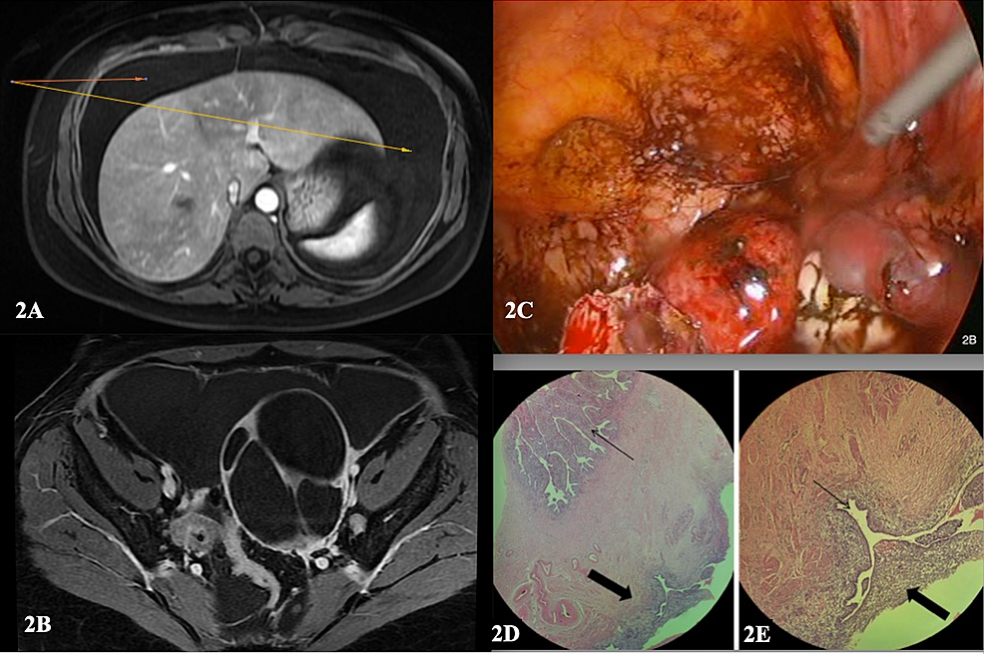
(2A) Axial Image - MRI demonstrating (arrows) gross ascites around liver, (2B) Axial image - MRI demonstrating complex left ovarian cyst with gross ascites surrounding cyst (2C) Extensive peritoneal and pelvic endometriosis. (2D) Micrographs demonstrating the histological features of endometriosis involving a segment of fallopian tube (FT). A. 200x. There is a focus of endometriosis (thick arrow) on the outer/serosal surface of the fallopian tube . The thin arrow highlights normal intra-luminal FT mucosa, (2E) 400x Higher magnification of the focus of endometriosis characterised by glandular spaces lined by endometrial epithelium (thin arrow) and endometrial-type stroma (thick arrow).

Histological analysis of post-operative specimen showed two fragments of tan and dark brown membranous tissue (cyst wall fragments) measuring 12.0cm x 2.1cm x 0.8cm and 11.1cm x 4.1cm x 0.3cm respectively, consistent with an endometriotic cyst. The 3.5cm x 2.8cm x 2.5cm segment of fallopian tube demonstrated extensive endometriosis (Figure 2). The specimen was negative for malignancy.

Abdominal and pelvic ultrasonography showed abdominal fluid and a 7cm left septate cyst. A magnetic resonance (MRI) scan of the abdomen and pelvis displayed normal appearances of the intra-abdominal organs. The pelvic scan showed 6.8cm x 9.5cm x 7.1cm multiloculated cystic mass in the left adnexa, with well-defined margins and no evidence of fat stranding or modularity (Figure 2). No fatty or calcific areas were seen within the mass. A large volume of ascites was present with absent peritoneal thickening (Figure 2). The posterior aspect of the mass abutted the distal left ureter causing external compression, resulting in mild left-sided hydronephrosis and left hydroureter. The uterus was retroverted but normal-sized, and the endometrial layer, uterine walls, cervix, right ovary and urinary bladder appeared normal. There was no evidence of inguinal or pelvic lymphadenopathy and otherwise unremarkable study.

Abdominocentesis was performed and six litres of ascites was drained. One litre of cloudy, amber peritoneal fluid was sent for analysis. Cytology showed mesothelial cells with reactive changes and no evidence of malignant cells. Biochemical analysis of the ascitic fluid revealed an albumin concentration of 1.7g/dL, total protein 2.5g/dL, lactate dehydrogenase (LDH) 184U/L and amylase 108U/L. Blood parameters were all normal with a CA-125 value of 89u/ml.

The diagnosis of ascites secondary to endometriosis was made. Patient was treated with GnRH subcutaneous injections monthly. The patient had to have repeated ascitic drainage every week for three weeks after her first dose. Each ascitic tap drained a total of 5.5 litres of serosanguinous fluid. The fluid continued to accumulate slowly but did not require drainage. This eventually resolved after her second dose of GnRH analogue. After the sixth dose, an ultrasound was done which showed an enlarged but otherwise normal left ovary, right polycystic ovary, no endometrial cysts, and no ascites. This patient was then placed on oral Dienogest 2mg daily with no recurrence for 2 years.

Case 3

A 33-year-old nulliparous woman of Afro Trinidadian descent with a history of recurrent ascites for 15 years, presented with progressive abdominal distension and dysmenorrhoea for two months. She has no known medical conditions, and her past surgical history is significant for an appendectomy performed 12 years ago. Previously, the patient had been extensively investigated for the source of her ascites at various health institutions, however, the cause remained uncertain. An ascitic tap done two years prior was also inconclusive. At that time, a biochemical analysis of the ascitic fluid revealed an albumin concentration of 3.6g/dL, protein 5.7g/dL, globulin 0.9g/dL, lactate dehydrogenase (LDH) 165U/L, and amylase 393U/L. In addition, her haematological, renal and liver panels were all within normal parameters. However, her serum albumin was 3.6g/dL indicating a serum-ascitic albumin gradient (SAAG) score of zero. Cytological analysis of the ascitic fluid revealed the absence of malignant cells.

At this presentation, abdominal and pelvic ultrasonography revealed the presence of abdominal and pelvic free fluid. Blood investigations were all within normal parameters; however, her CA-125 levels were elevated at 47.90 U/ml, thus raising the suspicion of malignancy. A computed tomography (CT) scan of the chest, abdomen and pelvis revealed a thick-walled cystic mass in the right adnexa measuring 5.2cm x 3.8cm x 4.8cm with a volume of 47.4cc. There was free fluid noted in the right adnexa extending to the pouch of Douglas. Features of malignancy and lymphadenopathy were absent, and the intra-abdominal viscera appeared unremarkable. An attempt was made to obtain another ascitic sample for testing, however, this was deemed unsuccessful.

**Figure 3:**
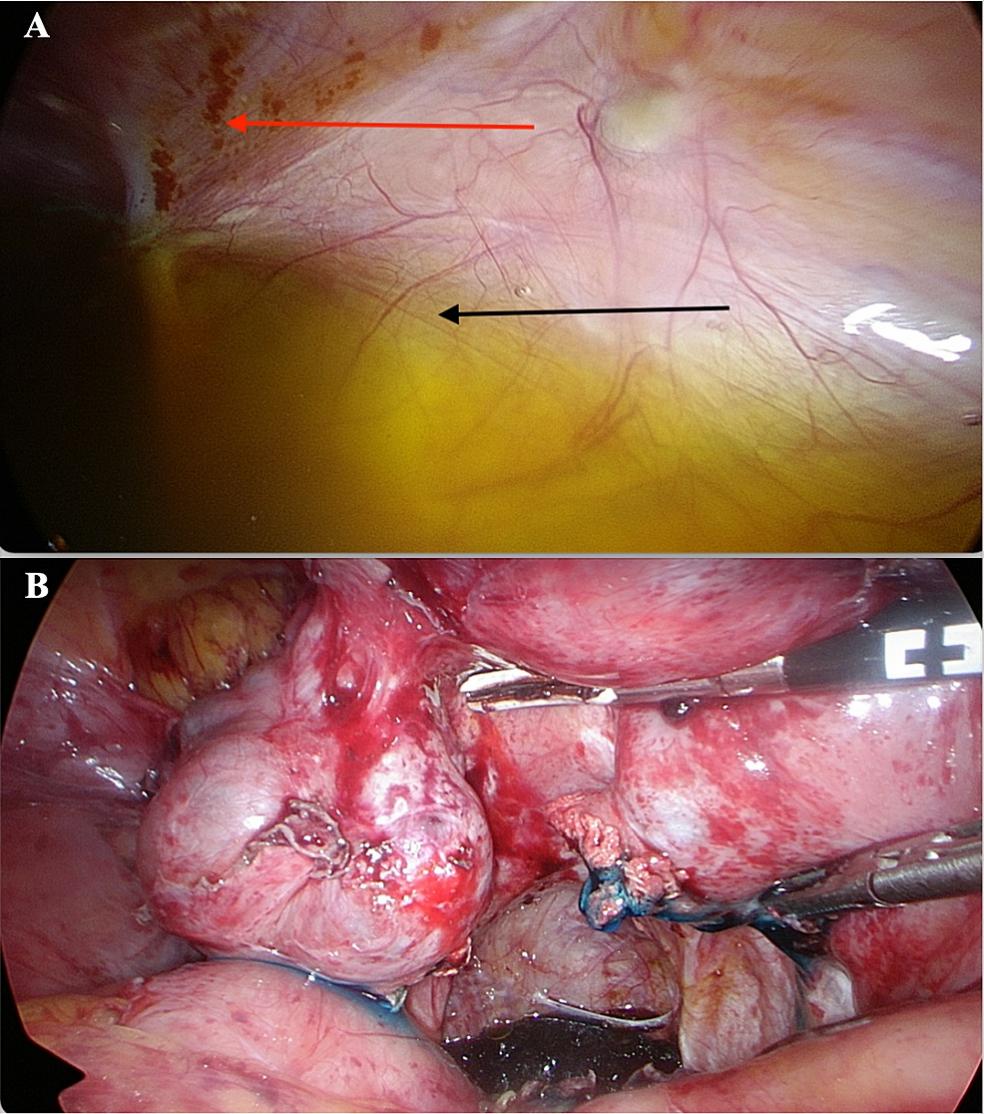
(A) Intra-operative photograph of ascites (Black Arrow) and endometriosis lesions (Red Arrow). (B) Extensive peritoneal endometriosis with blue dye seen, confirming tubal patency.

The patient was scheduled for a diagnostic laparoscopy. Intraoperatively, gross haemorrhagic ascites was noted with the presence of multiple foci of endometriosis (Figure 3). A right adnexal cystic mass was noted which was found to be an endometrioma. Cytological analysis of ascitic fluid taken intraoperatively revealed the presence of scant mesothelial cells, histiocytes and degenerate cells. Malignant cells were absent.

A diagnosis of ascites secondary to endometriosis was made and the patient was treated with the oral progestin Dienogest 2mg daily. At follow-up visit two, four and seven months later, the patient had no further abdominal distension, resolution of her dysmenorrhoea and no sonographic evidence of abdominal or pelvic free fluid.

## Discussion

Endometriosis is a significant cause of morbidity in the reproductive age group and diagnosed in up to 30% of women evaluated for infertility. The symptoms of endometriosis are directly related to the sites of implantation of ectopic endometrial tissue [8]. Women with deposits in the ovary and recto-vaginal pouch experience pelvic pain and deep dyspareunia whilst peritoneal deposits cause dense intra-abdominal adhesions resulting in distorted pelvic anatomy and infertility [4, 8]. Endometriosis can also present in the urinary tract, gastrointestinal tract, pleura and surgical scars which may lead to organ-specific symptoms [9]. Despite the numerous presentations of endometriosis, its association with ascites is rare.

Brews et al. described the first case of ascites related to endometriosis in 1954 and since then, approximately 60 cases have been described [10]. Moreover, such a case has rarely been described in the Caribbean literature. This clinical entity is prevalent in nulliparous women of African descent [11]. Though ascites may be associated with other gynaecological conditions such as ovarian cancer, Meig's syndrome, ruptured ovarian cysts and ectopic pregnancy, its relationship with endometriosis is important as it resembles malignancy [12].

When associated with ascites, patients experience abdominal distension, bloating, early satiety, dyspepsia, dyspnoea, weight loss and urinary urgency, frequency or incontinence [13]. Such features may be indistinguishable from ovarian malignancy [14]. A systematic review done by Gungor et al. in 2010 reported that ovarian malignancy was suspected in 52.4% of cases during diagnostic work-up [5]. Surgical assessment is therefore imperative for a conclusive diagnosis and to rule out an ovarian malignancy.

The pathogenesis of this condition remains unelucidated. Several theories attempt to explain the association of ascites with endometriosis. Magalhaes et al. hypothesised that peritoneal endometriotic deposits undergo cyclical bleeding resulting in fibrosis and inflammation, which produces a haemorrhagic exudate in the peritoneal cavity [15]. Another theory suggests that the rupture of endometriotic ovarian cysts result in the irritation of peritoneal surfaces which produces a reactive exudate and haemorrhagic ascites [3, 6, 15]. Regardless of its pathophysiology, this condition is characterised by high recurrence rates.

The diagnosis of ascites secondary to endometriosis is confirmed on surgical assessment. Currently, there are no pathognomonic biochemical markers for endometriosis [15]. Serum CA-125 concentration is a non-specific biomarker often elevated in both ovarian cancer and advanced endometriosis [14]. Thus, a considerable increase in CA-125 concentrations often mimic and increases the suspicion of malignancy. In this case, imaging and abdominal paracentesis is recommended. Pelvic ultrasonography may reveal the presence of abdominal and pelvic free fluid suggestive of massive ascites [13]. In addition, features of endometriosis like a fixed, retroverted uterus, hypoechoic lesions in comparison with the uterine myometrium and the ground glass appearance of an ovarian endometrioma may be present [16, 17].

Abdominal paracentesis is useful to identify the possible source of ascitic fluid. Macroscopic evaluation of ascitic samples produced by endometriosis may appear haemorrhagic or serosanguinous [15]. Cytological analysis often reveals the presence of hemosiderin-laden histiocytes and the absence of malignant cells [18]. Such findings are suggestive of haemorrhagic ascites and surgical histopathology is necessary to exclude the possibility of malignancy [19].

Biochemical analysis of ascitic fluid to differentiate among the common causes of ascites include a measurement of ascitic and serum albumin, protein, lactate dehydrogenase (LDH) and amylase [20]. Protein and LDH aid in the diagnosis of spontaneous bacterial peritonitis whereas the presence of amylase may indicate acute pancreatitis [20]. Calculation of the serum-ascites albumin gradient (SAAG) score differentiates ascites into an exudative versus transudative aetiology. Transudative ascites (SAAG score > 1.1mg/dL) is caused by cirrhosis, cardiac failure and portal vein thrombosis whilst exudative ascites (SAAG score < 1.1mg/dL) is the result of malignancy, tuberculosis, acute pancreatitis and very rarely, endometriosis [21]. The patient described in the third case had a SAAG score of zero indicating both the possibility of malignancy or severe endometriosis.

Diagnostic laparoscopy is the gold standard for diagnosing and staging endometriosis [22]. Findings at laparoscopy include massive haemorrhagic ascites, discrete endometriotic nodules, ovarian endometriomas and dense intraabdominal adhesions [22, 23]. ­Histological analysis of surgically resected tissue can exclude malignancy and definitively diagnose endometriosis with the presence of at least two of the following: endometrial glands, stroma or hemosiderin-laden histiocytes [24].

Once malignancy has been excluded, laparoscopy can offer initial surgical treatment in women wishing to preserve fertility. Such surgical techniques include aspiration of ascitic fluid, endometriotic lesion ablation or resection, and adhesiolysis [25-27]. However, these techniques are temporizing and the recurrence of both ascites and endometriosis is common [26, 28]. In 2001, Jones et al. reported that 63% of women experienced symptom relief with laparoscopic endometriotic lesion ablation, but recurrence occurred in 74% of patients by 73 months postoperatively [28].

Medical treatment is important to reduce recurrence [29]. Failure to bridge fertility sparing surgery with medical therapy or cessation of medical therapy cause recurrence of symptoms which lead to higher morbidity and increased infertility rates [30]. Medical treatment options include combined oral contraceptives (COC), progestins, GnRH analogues and danazol [5, 31]. New progestins are now recommended as first-line hormonal therapy in the management of endometriosis over COC [32]. Notably, Dienogest is a new fourth-generation progestin which offers an effective and tolerable alternative to GnRH analogues and definitive surgical intervention in the long-term treatment of endometriosis [33]. Daily administration of Dienogest 2mg following fertility sparing surgery is associated with lower recurrence rates of both ascites and symptoms of endometriosis [15, 31, 34]. Dienogest does not reduce estradiol concentrations to post-menopausal levels like GnRH analogues and hence can be used indefinitely until pregnancy is desired or side effects occur [34].

GnRH agonists such as leuprolide acetate, goserelin and nafarelin cause pituitary desensitisation to gonadotrophs and subsequent loss of ovarian steroidogenesis leading to the regression of endometrial implants [5, 35]. GnRH agonists cause a significant decrease in endometriosis symptoms and ascites, however, its continuous use is limited only up to six months [35, 36]. This is because the hypoestrogenic state simulate menopause and women experience bone loss, vaginal dryness and atrophy, hot flashes and dyslipidaemia with extended use [36]. Although GnRH agonists are effective and compare to that of definitive surgical intervention, it is limited by its cost and side effects [36]. Definitive surgical intervention in the form of bilateral salpingo-oophorectomy and hysterectomy in women who have completed their family is an effective treatment option with low rates of recurrence [5, 37, 38].

## Conclusions

In conclusion, recurrent massive ascites is a rare complication of endometriosis. Clinicians should be mindful of this unusual presentation as it mimics a gynaecological malignancy. In women wishing to preserve fertility, this condition can be successfully managed with hormonal therapy thus avoiding surgical menopause. Unfortunately, recurrence is common with the cessation of medical treatment. This case series highlights the rare presentation of advanced endometriosis and emphasizes the importance of medical treatment to curtail symptoms, reduce recurrence and preserve fertility.
